# Cutaneous Larvae Migrans Treated with a Single Dose of Ivermectin

**DOI:** 10.1155/2022/8215335

**Published:** 2022-11-16

**Authors:** Iris S. Harrison, Kiran Lukose, Bhagwan Dass, Nila S. Radhakrishnan

**Affiliations:** University of Florida College of Medicine, P.O. Box 100238, Gainesville, FL 32610, USA

## Abstract

Hookworm-related cutaneous larva migrans (HrCLM) is a clinical diagnosis based on a history of exposure to contaminated soil and is associated with a characteristic red serpiginous lesion that migrates within the *epidermis*. Our patient presented with a red, tortuous migratory rash with localized pruritus on the left plantar foot of 1-month duration. He lacked recent travel history outside the southeastern United States. Upon admission, he presented with peripheral blood eosinophilia, an uncommon feature of HrCLM. A single dose of ivermectin was sufficient for treatment, and symptoms resolved within 3 days. This case highlights the increased incidence of domestically acquired hookworm infections, explores this epidemiological shift, and emphasizes relevant differential diagnoses.

## 1. Introduction

An estimated 500 million individuals in developing tropical and subtropical countries are infected with hookworms [1]. Unlike the human hookworms, the dog hookworm, *Ancylostoma braziliense*, and cat hookworm, *A. caninum*, do not intentionally target humans. However, they can incidentally infect humans percutaneously, and this zoonotic parasitic infestation results in hookworm-related cutaneous larva migrans (HrCLM) [2]. HrCLM classically presents as an erythematous, slightly raised, serpiginous or linear skin lesion that migrates within the *epidermis*. The phenomenon is known as a “creeping eruption” or “creeping dermatitis” [3].

*A. braziliense* and *A. caninum* reside in the gastrointestinal tracts of dogs and cats. Their eggs are expelled in animal feces and deposited in the soil [2]. The consequent infective larvae can penetrate the corneal layer of the *epidermis* on any areas of exposed human skin that come into contact with contaminated soil. This typically occurs through the soles of individuals walking barefoot outside. Common sources of infection include beaches, sandboxes, and crawl spaces under dwellings, but any soil that may come in contact with animal droppings can be a potential reservoir [4]. The larvae produce hyaluronidase and a protease that allows their intradermal passage [3]. They cannot further develop in their incidental human host and are stuck migrating aimlessly within the *epidermis* until the larvae die. Consequently, HrCLM is innately self-limiting and resolves in as little as a few weeks to as long as a year [5].

HrCLM is one of the most common infectious diseases of international travelers [6]. Historically, it has been attributed to travel to tropical and subtropical developing countries of Asia, Africa, and the Americas [6]. However, *A. braziliense* and *A. caninum* are also endemic to the southeastern United States, notably along the Atlantic Ocean and Gulf of Mexico [7]. The increasing incidence of these infections in the southern United States may be a result of recent climate change, as these parasites thrive in warm, sandy conditions [8]. As cases of local soil transmission of hookworms rise, it is imperative that clinicians in the West be alert to identifying and differentiating this zoonotic parasite from other conditions with similar presentations, such as Löffler syndrome and DRESS syndrome, that bear considerably greater morbidity [9, 10].

## 2. Case Presentation

A well-appearing male in his twenties with no significant past medical history presented to the emergency department initially for an erythematous rash that tracked proximally up his left arm, as well as left arm numbness and paraesthesias, following trauma to his left hand 2 weeks prior. He was admitted to the hospital for possible cellulitis with acute lymphangitic spread.

During admission, the patient noted a second rash on the medial aspect of his left plantar foot that had appeared 1-month prior. The erythematous lesion was serpiginous, intensely pruritic, and frequently changed locations overnight. He tried topical antibiotic ointment and hydrogen peroxide on the area without improvement.

The patient lived at home with his dogs and stray cats. He was a roofer by trade and frequently came into contact with sand and soil while working with construction materials. He wore “flip-flops” sandals on occasion but denied walking outside barefoot. He also denied going to the beach, local water springs, or any recent travel. The patient did not endorse any fevers, chills, nausea, vomiting, shortness of breath, headaches, or changes in vision. He was uncertain of the last Tdap immunization and reported no IV drug use.

**Figure 1:**
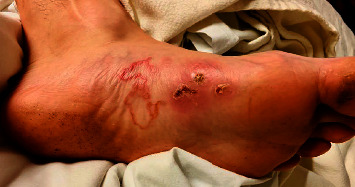
The site of the injury on the plantar surface of the foot displaying linear open bullae, serpiginous rash, and surrounding erythema.

On examination, multiple traumatic lesions with pustules and bullae were present on his hands bilaterally. The left hand displayed a large blood-filled blister on the medial wrist with surrounding erythema that radiated proximally toward his left arm. His left plantar foot exhibited an erythematous tortuous lesion with a migrating track adjacent to an approximately 4 cm patch of erythema with a central scab and bullae (Figure 1). All the other examinations were unremarkable.

On the day of admission, laboratory findings showed elevated white blood cells (15.3 × 10^9^/L; reference range 4.0–10 × 10^9^/L), C-reactive protein (22.69 mg/dL; reference range 0–5 mg/L), and erythrocyte sedimentation rate (32 mm/hour; reference range 0–10 mm/hour). His overall eosinophil count was within normal limits (0.14 × 10^9^/L; reference range 0.03–0.46 × 10^9^/L), but his eosinophils occupied a high proportion of total white cells (15%). No skin biopsy, stool testing, or chest X-ray was performed.

### 2.1. Differential Diagnosis

The differential diagnosis of a man in his late twenties presenting with a 1-month history of a migratory serpiginous rash with associated pruritus includes HrCLM, larva currens of strongyloidiasis, gnathostomiasis, and scabies.

The rash of HrCLM is red, well-defined, persistent, and slowly migrates at a rate of 1–2 cm/day [4]. When suspecting a HrCLM, the main condition that must be ruled out is larva currens, the migrating rash of the human nematode *Strongyloides stercoralis*. In comparison to HrCLM, the rash of larva currens is pink, less defined, evanescent, and rapidly migrates at a rate of 5–15 cm/hour. The lower trunk, buttocks, and thighs are commonly affected. Larva currens also presents with marked urticaria, systemic symptoms, GI manifestations, peripheral eosinophilia, and stool positive for larvae of *S. stercoralis* [11].

Gnathostomiasis is caused by the larvae of *Gnathostoma* spp. migrating through subcutaneous tissues. It can present as a creeping eruption with intermittent, migratory swellings of the trunk and upper extremities. However, gnathostomiasis is a food-borne parasite that often follows ingestion of raw or contaminated seafood or travel to endemic countries of Southeast Asia, Central, or South America [12].

Scabies represents the cutaneous manifestation of a *Sarcoptes scabiei* mite infection. Scabies presents as intensely pruritic linear or serpiginous burrows in multiple areas, commonly the interdigital spaces of the hand, wrists, intertriginous areas, and genitalia [13].

### 2.2. Treatment and Outcome

Our patient was diagnosed with hookworm-related cutaneous larva migrans (HrCLM) and treated with a single dose of ivermectin 200 *μ*g/kg.

The left-hand clinical picture of trauma and erythema quickly streaking up the left arm combined with a white blood cell count of 15.3 × 10^9^/L prompted a clinical suspicion of cellulitis with acute lymphangitic spread. The patient completed antibiotics and received a Tdap vaccine booster.

The patient's creeping dermatitis and pruritus of the left plantar foot resolved within 3 days. At that time, he was discharged to go home.

## 3. Discussion

Our case demonstrates a classic presentation of HrCLM acquired domestically in the southeastern United States. As our patient lacked recent travel history, he was likely exposed while wearing flip flops on the ground around his home that had been soiled by his dogs and cats. Alternatively, since he worked in roofing, he may have been exposed to contaminated soil while handling building materials on the job. Traditionally, HrCLM has been associated with travel to sandy beaches that are contaminated with the filariform larvae of animal feces. Indigenous cases of HrCLM in patients without a history of travel have been increasingly reported [14]. Much attention has been given to the effects of global warming on the incidence of human helminth infections [8]. Warmer climates and increased rainfall increase the tropical and subtropical zones on the global map and foster ideal conditions for the life cycle of *A. braziliense* and *A. caninum* [3]. Therefore, climate change may be contributing to an increased incidence of domestically acquired animal hookworm infections and should be a continued area of interest for researchers.

The diagnosis was made clinically. No skin biopsy or stool testing was performed. Skin biopsies are rarely diagnostic of HrCLM since the larvae are typically located 1–2 cm ahead of the visible lesion [4]. In our case, the classical clinical picture strongly suggested the diagnosis of HrCLM without the need for further testing. However, in cases with less clear clinical pictures, testing for *S. stercoralis* antibodies and larvae in stool would be helpful to rule out the diagnosis of larva currens of strongyloidiasis.

Our patient is notable in that he presented with peripheral eosinophilia of 15%. In a study of 98 German travelers with HrCLM, 20% of the 40 individuals tested presented with eosinophilia >7% [15]. While our patient presented with peripheral eosinophilia, he did not report any respiratory symptoms or display any signs of pulmonary distress, so a chest X-ray was not performed. However, HrCLM can present in a self-limited disseminated form called Löffler syndrome (pulmonary eosinophilia), characterized by an immediate onset of peripheral eosinophilia, transient pulmonary infiltrates, and respiratory symptoms [9]. Löffler syndrome can be seen following infection by hookworms as well as *Ascaris lumbricoides* and *S. stercoralis*. In cases of Löffler syndrome, the differential diagnosis must include drug reaction with eosinophilia and systemic symptoms (DRESS) syndrome with pulmonary manifestation, as these two conditions can present identically [10]. While Löffler syndrome is mild and self-limited, DRESS syndrome is severe and potentially fatal. DRESS syndrome is a hypersensitivity reaction that occurs 2–8 weeks after drug exposure (often allopurinol, anticonvulsants, sulfonamides, dapsone, and other antimicrobials) with a variable clinical presentation that can include high fever, malaise, skin eruption, eosinophilia, lymphadenopathy, and visceral organ involvement (commonly liver, kidney, and lungs) [16]. The skin rash is often the first clear manifestation of DRESS syndrome and often presents as a symmetric maculopapular rash involving >50% of the trunk and extremities that later coalesces with erythema [17]. Mucosal involvement, pruritus, and facial edema may also occur [17]. Pulmonary involvement is rare but signifies a greater severity of the disease. When pulmonary involvement does occur, it most commonly presents with cough, shortness of breath, and interstitial infiltrates on imaging [10]. Due to its nonspecific presentation, DRESS is referred to as a “great mimicker” and is a diagnosis of exclusion [10]. With a mortality rate of 10%, DRESS syndrome is an important differential diagnosis to consider in any patient presenting with a rash [17].

Although HrCLM is self-limiting, pharmacological treatment of choice includes oral ivermectin 200 *μ*g/kg once daily for 1–2 days or oral albendazole 400–800 mg once daily with a fatty meal for 3 days [4]. A similar case of HrCLM acquired in Florida was successfully treated with two doses of ivermectin [18]. However, a single dose of oral ivermectin is sufficient for patients with HrCLM with creeping dermatitis, as in the case of our patient [19]. Ivermectin acts on ligand-gated chloride channels in nematodes to induce paralysis and impede feeding and fertility [20]. Inconsistent results have been reported in patients treated with topical ivermectin 1% cream twice daily for two weeks [18, 21, 22]. Interestingly, a recent report observed eradication of HrCLM in a patient treated with a low-dose ivermectin 0.1% cream twice daily for two weeks [23]. Additionally, topical thiabendazole 10–15% ointments and albendazole may provide an alternative, albeit less potent, treatment [15].

## 4. Conclusion

In summary, HrCLM is a clinical diagnosis classically associated with international travel to tropical areas. However, cases of indigenous HrCLM are increasingly reported. This change in epidemiology may be partially attributed to changes in climate that have shifted the tropical landscape. Therefore, clinicians must keep this diagnosis as well as other similar clinical presentations in mind. If diagnosed promptly, this skin disease responds very well to pharmacological therapy, and often a single dose of ivermectin adequately resolves the infection.

## Data Availability

No data were used to support this study.

## References

[1] Loukas A., Hotez P. J., Diemert D. (2016). Hookworm infection. *Nature Reviews Disease Primers*.

[2] Bowman D. D., Montgomery S. P., Zajac A. M., Eberhard M. L., Kazacos K. R. (2010). Hookworms of dogs and cats as agents of cutaneous larva migrans. *Trends in Parasitology*.

[3] Heukelbach J., Feldmeier H. (2008). Epidemiological and clinical characteristics of hookworm-related cutaneous larva migrans. *The Lancet Infectious Diseases*.

[4] Hochedez P., Caumes E. (2007). Hookworm-related cutaneous larva migrans. *Journal of Travel Medicine*.

[5] Caumes E. (2000). Treatment of cutaneous larva migrans. *Clinical Infectious Diseases*.

[6] Herbinger K. H., Beissner M., Berens-Riha N. (2016). Spectrum of imported infectious diseases: a comparative prevalence study of 16, 817 German travelers and 977 immigrants from the tropics and subtropics. *The American Journal of Tropical Medicine and Hygiene*.

[7] Starr M. C., Montgomery S. P. (2011). Soil-transmitted helminthiasis in the United States: a systematic review—1940–2010. *The American Journal of Tropical Medicine and Hygiene*.

[8] Blum A. J., Hotez P. J. (2018). Global worming: climate change and its projected general impact on human helminth infections. *PLoS Neglected Tropical Diseases*.

[9] Podder I., Chandra S., Gharami R. C. (2016). Loeffler’s syndrome following cutaneous larva migrans: an uncommon sequel. *Indian Journal of Dermatology*.

[10] Taweesedt P. T., Nordstrom C. W., Stoeckel J., Dumic I. (2019). Pulmonary manifestations of drug reaction with eosinophilia and systemic symptoms (DRESS) syndrome: a systematic review. *BioMed Research International*.

[11] Jourdan P. M., Lamberton P. H. L., Fenwick A., Addiss D. G. (2018). Soil-transmitted helminth infections. *The Lancet*.

[12] Herman J. S., Chiodini P. L. (2009). Gnathostomiasis, another emerging imported disease. *Clinical Microbiology Reviews*.

[13] Thomas C., Coates S. J., Engelman D., Chosidow O., Chang A. Y. (2020). Ectoparasites: scabies. *Journal of the American Academy of Dermatology*.

[14] Giudice P., Hakimi S., Vandenbos F., Magana C., Hubiche T. (2019). Autochthonous cutaneous larva migrans in France and Europe. *Acta Dermato-Venereologica*.

[15] Jelinek T., Maiwald H., Nothdurft H. D., Löscher T. (1994). Cutaneous larva migrans in travelers: synopsis of histories, symptoms, and treatment of 98 patients. *Clinical Infectious Diseases*.

[16] Kardaun S. H., Sekula P., Valeyrie-Allanore L. (2013). Drug reaction with eosinophilia and systemic symptoms (DRESS): an original multisystem adverse drug reaction. Results from the prospective RegiSCAR study. *British Journal of Dermatology*.

[17] Chen Y. C., Chiu H. C., Chu C. Y. (2010). Drug reaction with eosinophilia and systemic symptoms: a retrospective study of 60 cases. *Archives of Dermatology*.

[18] Magri F., Chello C., Pranteda G., Pranteda G. (2019). Complete resolution of cutaneous larva migrans with topical ivermectin: a case report. *Dermatologic Therapy*.

[19] Vanhaecke C., Perignon A., Monsel G., Regnier S., Bricaire F., Caumes E. (2014). The efficacy of single dose ivermectin in the treatment of hookworm related cutaneous larva migrans varies depending on the clinical presentation. *Journal of the European Academy of Dermatology and Venereology*.

[20] Laing R., Gillan V., Devaney E. (2017). Ivermectin - old drug, new tricks?. *Trends in Parasitology*.

[21] Gelmetti C., Brena M., Veraldi S. (2019). Hookworm-related cutaneous larva migrans of the penis successfully treated with topical ivermectin. *Pediatric Dermatology*.

[22] Veraldi S., Angileri L., Parducci B. A., Nazzaro G. (2017). Treatment of hookworm-related cutaneous larva migrans with topical ivermectin. *Journal of Dermatological Treatment*.

[23] Rodenas-Herranz T., Linares-Gonzalez L., Martinez-Velez V., Ruiz-Villaverde R. (2020). Hookworm-related cutaneous larva migrans successfully treated with topical ivermectin 0.1. *Dermatologic Therapy*.

